# Online mouse cursor trajectories distinguish phonological activation by linguistic and nonlinguistic sounds

**DOI:** 10.3758/s13423-022-02153-6

**Published:** 2022-07-26

**Authors:** Anuenue Kukona, Adrian Jordan

**Affiliations:** 1 Division of Psychology, De Montfort University, Leicester, UK; 2 Present Address: School of Human Sciences, University of Greenwich, Old Royal Naval College, Park Row, SE10 9LS London, UK

**Keywords:** Cohort competition, Environmental sounds, Mouse cursor tracking, Phonological competition

## Abstract

Four online mouse cursor tracking experiments (total *N* = 208) examined the activation of phonological representations by linguistic and nonlinguistic auditory stimuli. Participants hearing spoken words (e.g., “bell”) produced less direct mouse cursor trajectories toward corresponding pictures or text when visual arrays also included phonologically related competitors (e.g., belt) as compared with unrelated distractors (e.g., hose), but no such phonological competition was observed during environmental sounds (e.g., the ring of a bell). While important similarities have been observed between spoken words and environmental sounds, these experiments provide novel mouse cursor evidence that environmental sounds directly activate conceptual knowledge without needing to engage linguistic knowledge, contrasting with spoken words. Implications for theories of conceptual knowledge are discussed.

Conceptual knowledge is closely linked to linguistic knowledge. For example, words are assumed to “reveal the stock of basic concepts” throughout the cognitive science literature (e.g., for critique, see Malt et al., 2015, p. 294). However, to what degree is the retrieval of conceptual knowledge independent of the retrieval of linguistic knowledge? The current research examined environmental sounds—nonlinguistic auditory stimuli that are produced by everyday entities and events (e.g., the ring of a bell). The current aim was to examine whether nonlinguistic sounds engage phonological representations like spoken words, supporting the close interaction of conceptual and linguistic knowledge.

In contrast to spoken words, environmental sounds lack hierarchical linguistic structure (e.g., phonology; Ballas & Howard, 1987; Gaver, 1993). Nevertheless, surprising parallels have been observed between the two. For example, not only do environmental sounds prime spoken words (Van Petten & Rheinfelder, 1995), but both types of auditory stimuli can prime corresponding pictures (Chen & Spence, 2011, 2018a, 2018b; Lupyan & Thompson-Schill, 2012), facilitate visual search for corresponding pictures (Iordanescu et al., 2011) and draw fixations to semantically related pictures (Bartolotti et al., 2020; Toon & Kukona, 2020). This behavioural evidence highlights the close link between both types of auditory stimuli and conceptual knowledge. Likewise, overlapping cortical regions are activated when participants match environmental sounds and spoken words to pictures (Dick et al., 2007), a related N400 response is elicited by pictures (mis)matching both types of auditory stimuli (Cummings et al., 2006) and individuals with aphasia show correlated impairments across both types of auditory stimuli (Saygın et al., 2003). This neurobiological evidence suggests that shared neural resources are recruited by linguistic and nonlinguistic auditory stimuli alike. Finally, while environmental sounds and spoken words are not processed identically, they are often distinguished quantitatively rather than qualitatively (e.g., both environmental sounds and spoken words were observed to prime corresponding pictures but the latter did so more; Lupyan & Thompson-Schill, 2012).

Underpinning these parallels, recent evidence suggests that environmental sounds may activate phonological representations like spoken words. In the case of spoken words, Allopenna et al. (1998) found that participants hearing “beaker” fixated a picture of a phonologically related beetle more than an unrelated carriage, reflecting the activation of (e.g., competing) phonological representations during language processing. In the case of environmental sounds, Bartolotti et al. (2020) observed closely related phonological competition: participants hearing either the ticking of a clock or “clock” fixated a picture of a phonologically related cloud more than an unrelated light bulb during both types of auditory stimuli. Perhaps unsurprisingly, these environmental sounds and spoken words were not processed identically; rather, their time courses differed. Nevertheless, these findings suggest that phonological representations are activated by linguistic and nonlinguistic auditory stimuli alike, highlighting the close interaction between both types of auditory stimuli and phonological representations, and supporting the close interaction of conceptual and linguistic knowledge. Note that here and throughout, we emphasize phonology, which is typical of the literature and reflects the focus on speech in studies (e.g., that make use of the visual world paradigm) like Allopenna et al. (1998) and Bartolotti et al. (2020). However, phonological competitors in alphabetic languages like English are often orthographic competitors, too, and thus it is possible that this competition was underpinned by linguistic knowledge spanning orthography alongside phonology.

However, recent findings also raise important questions about these insights. Marian et al. (2021) added a subsequent retrieval phase to Bartolotti et al.’s (2020) task and observed phonological competition (i.e., in recognition accuracy) for spoken words, but not environmental sounds. Relatedly, Kukona (2021) examined contextual influences and observed phonological competition when participants heard environmental sounds interleaved among spoken words, but not when they only heard environmental sounds. Moreover, as a point of comparison, semantic competition (e.g., activation of a semantically related bone when hearing barking) has been observed consistently across studies (e.g., Bartolotti et al., 2020; Marian et al., 2021; Toon & Kukona, 2020), unlike phonological competition. Rather, these findings may support Chen and Spence (2011, 2018a), who hypothesized that environmental sounds directly activate conceptual knowledge without needing to engage linguistic knowledge, contrasting with spoken words.

## The current study

Cognition unfolds over time. Thus, continuous measures of behaviour are essential for capturing underlying time course dynamics. Mouse cursor tracking methods provide a time course record of participants’ motor responses as they engage in cognitive processing. An important advantage of this method is its sensitivity to continuous behavioural dynamics (e.g., see Freeman et al., 2011; Magnuson, 2005). For example, Spivey et al. (2005) found that participants hearing spoken words (e.g., “candle”) produced less direct mouse cursor trajectories toward corresponding pictures when visual arrays also included phonological competitors (e.g., candy) as compared with unrelated distractors (e.g., jacket). In other words, participants’ trajectories were attracted to phonological competitors. Like Allopenna et al. (1998), these findings provide evidence for phonological competition. However, because participants can only fixate one point in space at any point in time, eye movement evidence for continuous dynamics typically requires, and might be an artefact of, aggregating across discrete (i.e., fixation) behaviours. In contrast, mouse cursor tracking captures truly continuous behavioural dynamics.

**Fig. 1:**
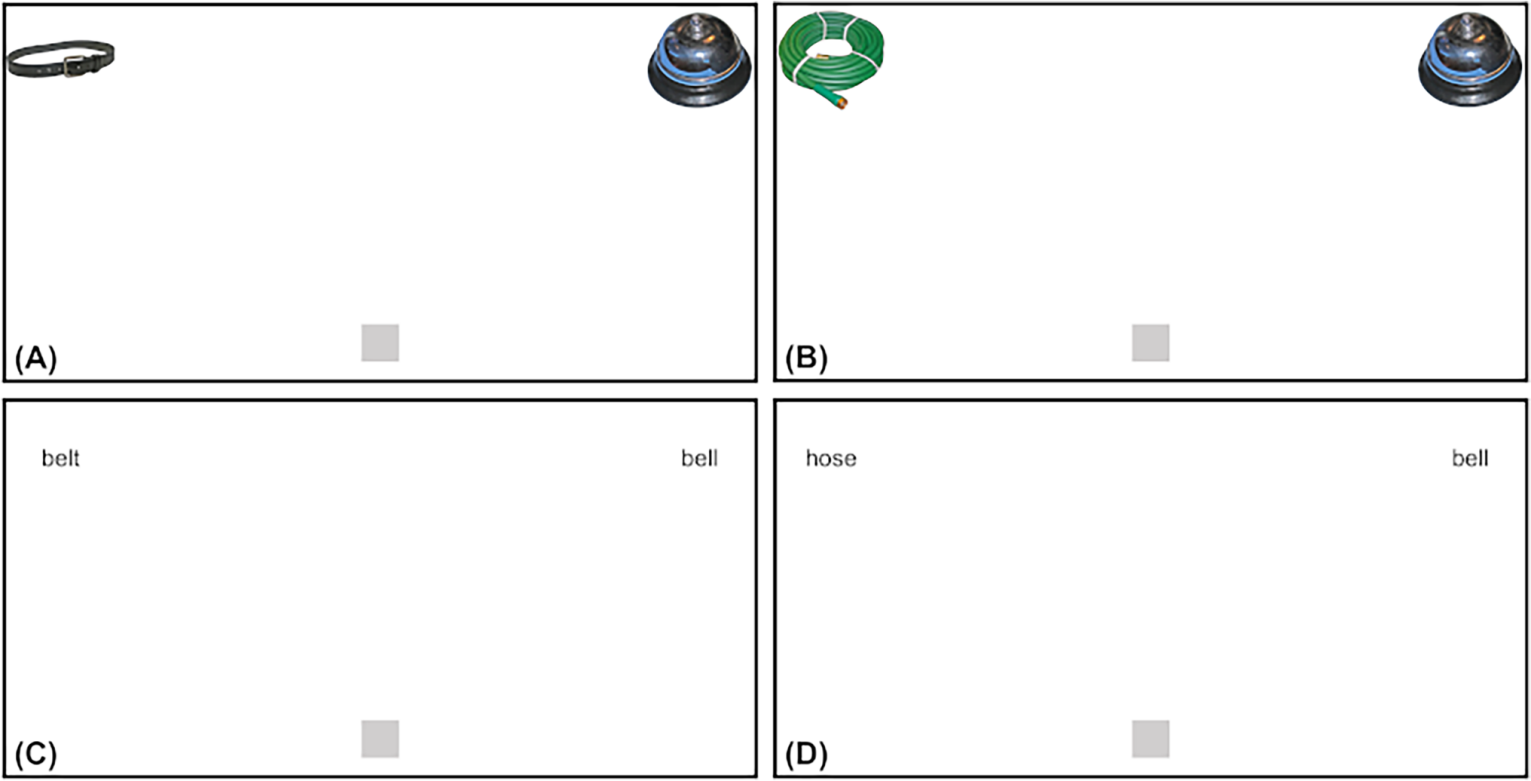
Example competitor (**a**) and distractor (**b**) picture arrays from Experiments 1 and 2, and competitor (**c**) and distractor (**d**) text arrays from Experiments 3 and 4, for the target “bell”. The competitor arrays included the phonologically (i.e., cohort) related belt and the distractor arrays included the unrelated hose

The current study exploited the sensitivity of mouse cursor tracking to continuous behavioural dynamics to examine whether phonological representations are activated by environmental sounds like spoken words. In Experiments 1 and 2, participants heard spoken words like “bell” or environmental sounds like the ring of a bell, respectively, while viewing visual arrays like Fig. 1a or Fig. 1b with pictures of a bell and phonologically (i.e., cohort) related belt or unrelated hose. Motivated by the (i.e., linguistic) contextual influences observed by Kukona (2021), Experiments 3 and 4 used arrays with written text rather than pictures, providing a visual linguistic context that may boost phonological influences. Building on Allopenna et al. (1998), Huettig and McQueen (2007) and McQueen and Viebahn (2007) similarly observed phonological competition when participants heard spoken words and viewed text rather than pictures. In Experiments 3 and 4, participants heard the same auditory stimuli from Experiments 1 and 2, respectively, while viewing visual arrays like Fig. 1c or Fig. 1d with text. If environmental sounds engage linguistic knowledge like spoken words, greater attraction to phonological competitors than to unrelated distractors was expected across all experiments.

## Method

### Participants

Fifty-two native English speakers from the USA with normal or corrected-to-normal vision and hearing were recruited through Prolific (https://www.prolific.co) to participate in each experiment. The samples enabled detection of a two-level within-participants effect size of *d*_z_ = 0.40 (power = .80, alpha = .05), which reflects an estimated average for psychological research (e.g., Brysbaert, 2019). In Experiment 1, one participant withdrew and another was excluded who used a touchscreen (i.e., as reflected in concentrated starting/ending coordinates); 50 participants were included in the analyses (age *M* = 36.08, *SD* = 11.61, 2 unreported; 23 female, 27 male). In Experiment 2, one participant withdrew and two others were excluded who either used a touchscreen or returned trajectories outside the visual array; 49 participants were included in the analyses (age *M* = 36.31, *SD* = 11.09; 23 female, 26 male). In Experiment 3, one participant was excluded who returned trajectories outside the visual array; 51 participants were included in the analyses (age *M* = 34.96, *SD* = 12.87, 3 unreported; 18 female, 32 male, one other). In Experiment 4, 52 participants were included in the analyses (age *M* = 34.40, *SD* = 12.38, 2 unreported; 20 female, 32 male).

### Design

Within each experiment, visual stimulus type (competitor and distractor) was manipulated within participants. Across experiments, auditory stimulus type (spoken word and environmental sound) and array type (picture and text) were manipulated. Experiments 1 and 2 used picture arrays, while Experiments 3 and 4 used text arrays, and Experiments 1 and 3 presented spoken words, while Experiments 2 and 4 presented environmental sounds.

### Norming

The names of the environmental sounds were normed in a separate study on Qualtrics (https://www.qualtrics.com). Fifteen participants were recruited through Prolific. Participants heard 56 environmental sounds. The environmental sounds were from Freesound (https://www.freesound.org) and corresponded to pictures from BOSS. The auditory files were converted to MP3s and their amplitudes were normalized. Participants were instructed to identify each auditory stimulus as briefly and unambiguous as possible by responding with only one name, the first that came to mind. As an attention check, participants also heard four words, which they were instructed to identify. The order of the auditory stimuli was randomized.

The proportion of responses that began with a name agreeing with the corresponding picture was computed for each environmental sound. For example, responses of “dog” and “dog barking” agreed with dog, but “barking dog” did not. Thirty environmental sounds with agreements ≥0.60 were included in the experiments (*M* = 0.80, *SD* = 0.12), which are reported in Table 2 in the Appendix.

### Materials

Thirty stimulus sets were assembled, which each included an environmental sound from the norming as a target (e.g., bell), a phonologically (i.e., cohort) related competitor (e.g., belt) and an unrelated distractor (e.g., hose). Competitors and distractors were rotated across targets, counterbalancing extraneous properties. Latent semantic analysis (i.e., cosines; Landauer & Dumais, 1997) revealed that competitors (*M* = 0.09, *SD* = 0.10) and distractors (*M* = 0.09, *SD* = 0.09) did not differ in their semantic relatedness with targets, *t*(28) = 0.03, *p* = .97 (cosines were unavailable for chainsaw). Visual arrays were assembled for each set either using pictures from BOSS (Fig. 1a–b) or corresponding text (Fig. 1c–d). Visual arrays used normalized coordinates (e.g., due to variation in participants’ screen resolutions, aspect ratios) ranging from −1 to 1. Coordinates for the left bottom of the visual array were (−1, −1), centre were (0, 0) and right top were (1, 1). Pictures were 0.3 × 0.6 centred at (±0.85, 0.70). Pictures were square for a 2:1 aspect ratio and stretched for others. Text was lower case Arial height 0.05 centred at (± 0.85, 0.70). Alongside the environmental sounds, corresponding target words were recorded by a male native speaker of American English. Environmental sounds (*M* = 4.66 seconds, *SD* = 3.33) were significantly longer in duration than spoken words (*M* = 0.86 seconds, *SD* = 0.18), *t*(29) = −6.39, *p* < .001.

Four counterbalanced lists were created by rotating the targets through the competitor/distractor conditions and left/right presentations in a Latin square. Each list included all 30 targets, one half presented with competitors and the other half presented with distractors, and one half presented on the left and the other half presented on the right of the visual array.

### Procedure

The experiments were created in PsychoPy (Peirce et al., 2019), and participants took part on Pavlovia (https://www.pavlovia.org). The experiments used static start and click response procedures without deadlines, there were no practice trials, and feedback was not provided (e.g., see Schoemann et al., 2021). Participants began each trial by clicking on an icon at the bottom of the screen (0, −0.85) and previewed the visual array for 0.50 seconds before hearing the auditory stimulus. Participants were instructed to use a computer mouse (e.g., rather than touchscreen) to click on the visual stimulus that corresponded to the auditory stimulus, which ended the trial. The order of trials was randomized.

### Analysis

Reaction times (RTs) were measured from trial onset. Log RTs were analyzed alongside mouse cursor trajectories, such that the latter provided important insight into the former (e.g., revealing whether RTs were slowed due to attraction to competitors). Inaccurate trials and trials with log RTs more than 2.5 standard deviations above the global mean were excluded from the analyses of RTs and trajectories. Left/right presentations of targets were combined by inverting the horizontal axis in the former. Following Spivey et al. (2005), trajectories across the visual array were aggregated by dividing each trial (e.g., which varied in duration) into 101 normalized time slices. To capture attraction to nontargets, the maximum signed deviation (MD) of each within-trial trajectory from the line connecting its starting and ending coordinates was computed. Thus, larger MDs were predicted for competitors than distractors (i.e., alongside slower RTs for these larger deviations), reflecting greater attraction to the former than latter. In addition, mean signed deviations across time (i.e., from the line connecting the start and end of each within-trial trajectory) were computed in 0.10-second time slices. Trial-level log RTs and MDs were submitted to linear mixed effects models with a deviation coded fixed effect of visual stimulus type (competitor = −0.5, distractor = 0.5) and random intercepts and slopes by participants and items. Maximal models were simplified by removing correlations among random effects or random slopes when there were issues with fit. Models were run in R using lme4 (Bates et al., 2015) and lmerTest (Kuznetsova et al., 2017). The R code is available in OSF. Given established and widely adopted methods for computing Bayes factor (BF) and effect size (*d*_x_) estimates for *t*-tests comparisons, log RTs and MDs were also aggregated by participants and submitted to participant analyses.

## Results

### Experiment 1

**Fig. 2:**
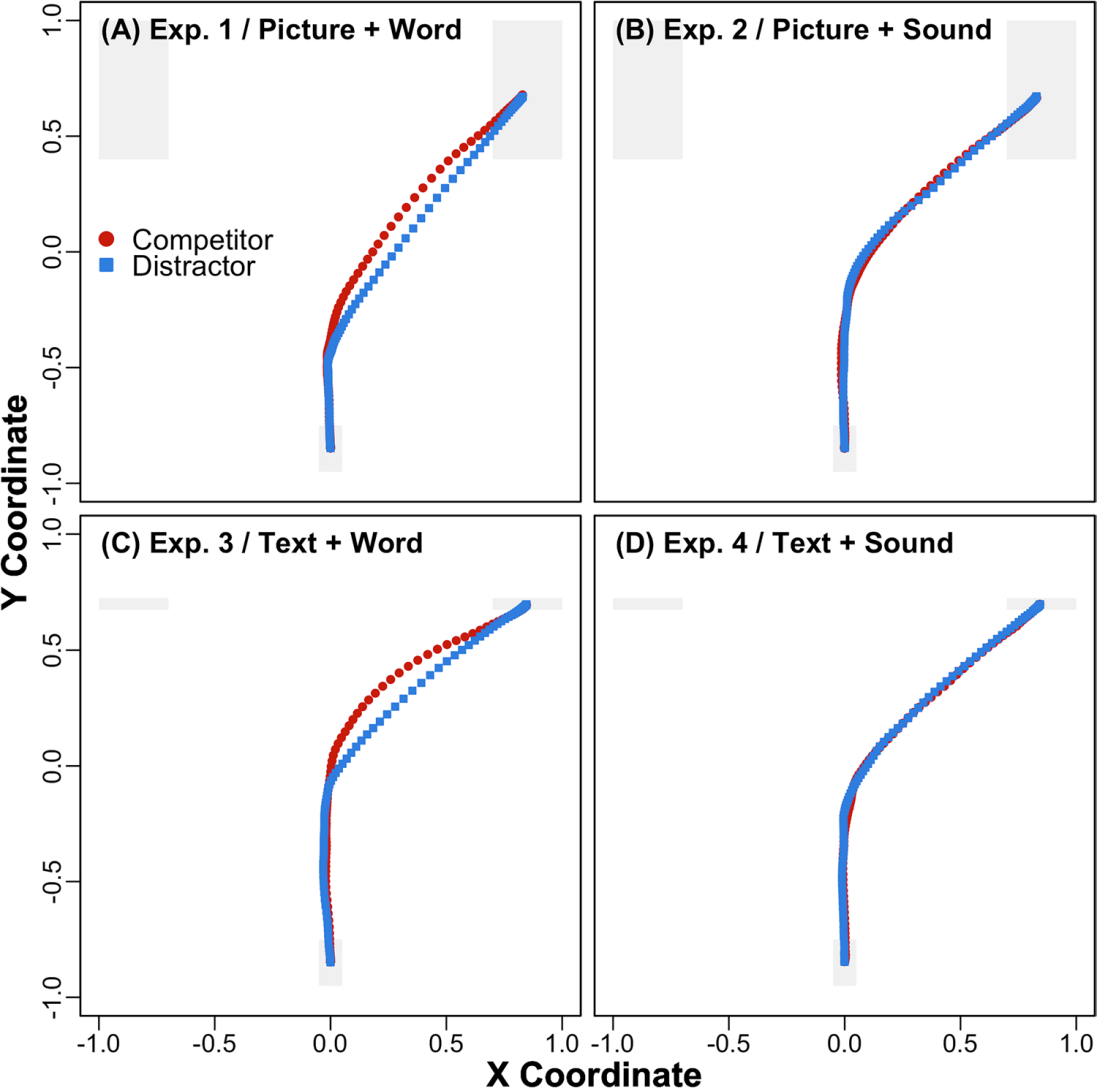
Time-normalized mean trajectories across the phonological competitor versus unrelated distractor arrays in Experiments 1–4 (**a–d**). Participants viewed picture arrays in Experiments 1 (**a**) and 2 (**b**) and text arrays in Experiments 3 (**c**) and 4 (**d**). Participants heard spoken words in Experiments 1 (**a**) and 3 (**c**) and environmental sounds in Experiments 2 (**b**) and 4 (**d**). Targets are plotted on the right and nontargets on the left

**Fig. 3:**
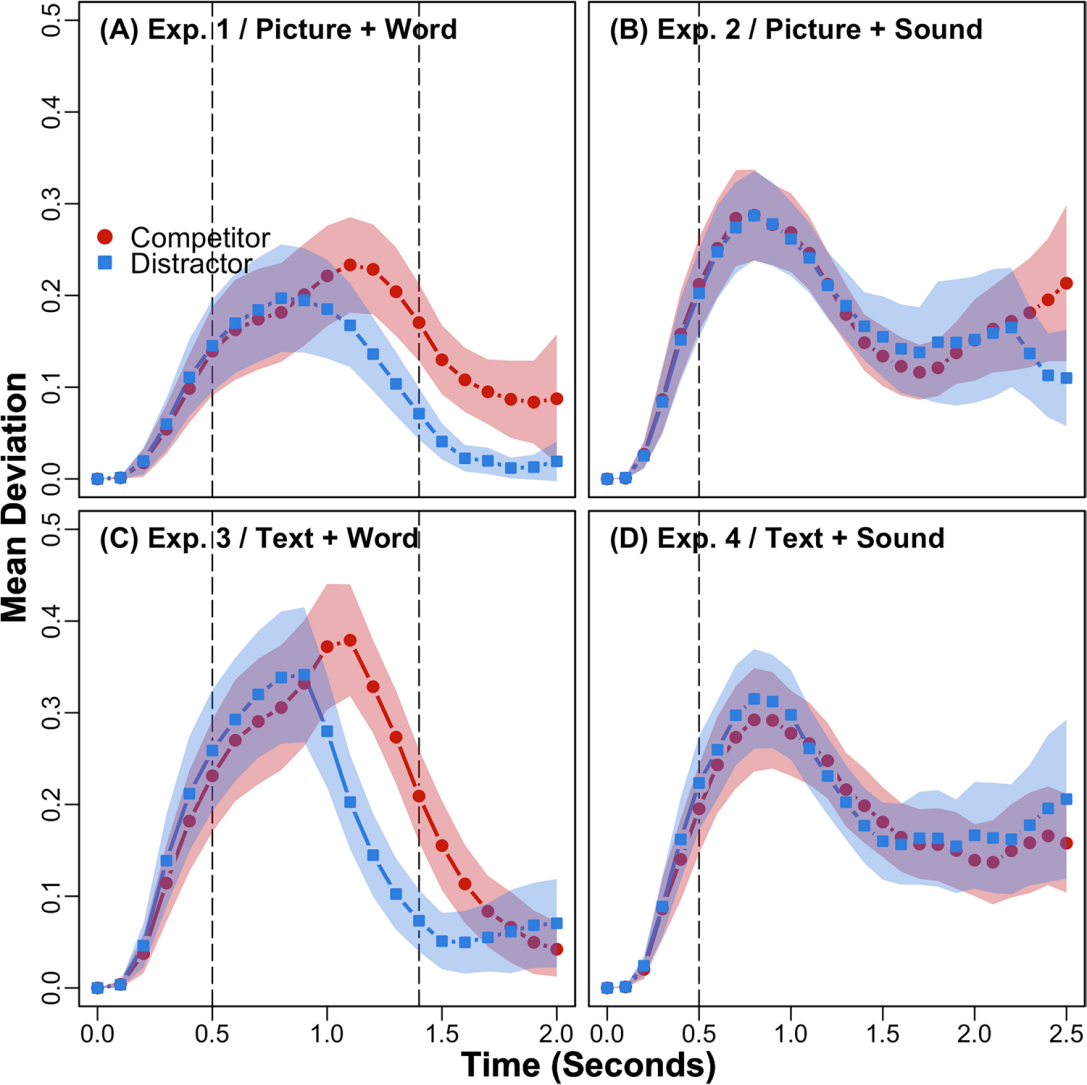
Mean deviations from the line connecting the start and end of each within-trial trajectory for phonological competitor versus unrelated distractor arrays in Experiments 1–4 (**a–d**). The plots span trial onset to +2 seconds for spoken words and +2.5 seconds for environmental sounds, reflecting their maximal approximate mean RTs. Shaded bands show 95% *CIs*, and vertical lines show onsets and mean offsets for the auditory stimuli (the latter are outside the plotted range for environmental sounds)

Participants heard spoken words while viewing picture arrays in Experiment 1. Accuracy was high across competitor (*M* = 98.40%, *SD* = 4.77) and distractor (*M* = 99.33%, *SD* = 2.78) conditions, and 2.29% of trials were above the RT threshold (4.24 seconds). Mean trajectories across the visual array and mean deviations across time are plotted in Figs. 2a and 3a. Mean RTs and MDs are reported in Table 1. RTs were significantly longer in the competitor than distractor condition, *Est*. = −0.03, *SE* = 0.01, *t*(30.68) = −2.62, *p* < .05. MDs were also significantly more attracted to competitors than distractors, *Est*. = −0.10, *SE* = 0.03, *t*(27.77) = −3.44, *p* < .01. Finally, Bayes’ factor analyses (i.e., BF_10_ reflects the inverse of BF_01_, supporting H_1_ over H_0_) provided strong (BF_10_ > 10 for RTs) to decisive (BF_10_ > 100 for MDs) support for these differences (see Table 1). Conceptually replicating Spivey et al. (2005), and consistent with Allopenna et al. (1998), these results support the activation of phonological competitors during language processing.

**Table 1:** Reaction times in seconds (RT) and maximum deviations (MD) by experiment for phonological competitor versus unrelated distractor arrays

DV	Competitor *M* (*SD*)	Distractor *M* (*SD*)	*t*	*p*	*d*_x_	BF_01_
Exp. 1 / Picture + Word (*N* = 50)						
*RT*	1.96 (0.46)	1.89 (0.45)	3.56	<.001	0.50	0.03
*MD*	0.41 (0.23)	0.31 (0.21)	6.29	<.001	0.89	0.00
Exp. 2 / Picture + Sound (*N* = 49)						
*RT*	2.01 (0.37)	1.99 (0.43)	0.92	.36	0.13	4.34
*MD*	0.47 (0.21)	0.47 (0.21)	0.33	.74	0.05	6.12
Exp. 3 / Text + Word (*N* = 51)						
*RT*	2.01 (0.43)	1.93 (0.48)	4.84	<.001	0.68	0.00
*MD*	0.61 (0.26)	0.50 (0.31)	3.61	<.001	0.51	0.03
Exp. 4 / Text + Sound (*N* = 52)						
*RT*	2.59 (0.74)	2.45 (0.71)	2.78	<.01	0.39	0.21
*MD*	0.48 (0.25)	0.50 (0.24)	-0.75	.46	0.10	5.07

Alongside means and standard deviations, participants *t*-test analyses, effect sizes (*d*_x_), and Bayes factors supporting H_0_ over H_1_ (BF_01_) are reported

### Experiment 2

Experiment 2 was identical to Experiment 1, but participants heard environmental sounds rather than spoken words while viewing picture arrays. Accuracy was high across competitor (*M* = 99.46%, *SD* = 2.29) and distractor (*M* = 99.46%, *SD* = 1.84) conditions, and 1.23% of trials were above the RT threshold (4.74 seconds). Mean trajectories and deviations are plotted in Figs. 2b and 3b. Mean RTs and MDs are reported in Table 1. Neither RTs, *Est*. = −0.01, *SE* = 0.02, *t*(27.58) = −0.79, *p* = .44, nor MDs, *Est*. = 0.00, *SE* = 0.03, *t*(28.22) = −0.13, *p* = .90, differed significantly between competitor and distractor conditions. Bayes’ factor analyses also provided substantial support (BF_01_ > 3) for these null effects (see Table 1). Finally, a trial-level mixed-effects model assessing the impact of experiment / auditory stimulus type (1 / spoken word = −0.5; 2 / environmental sound = 0.5), visual stimulus type and their interaction on MDs in picture arrays revealed a significant interaction between these fixed effects, *Est*. = 0.10, *SE* = 0.03, *t*(2733.75) = 3.60, *p* < .001. In contrast to Bartolotti et al. (2020), these results suggest that phonological competitors are not activated during environmental sound processing.

### Experiment 3

Participants heard spoken words while viewing text arrays in Experiment 3. Accuracy was high across competitor (*M* = 97.91%, *SD* = 4.11) and distractor (*M* = 99.87%, *SD* = 0.93) conditions, and 1.39% of trials were above the RT threshold (4.16 seconds). Mean trajectories and deviations are plotted in Figs. 2c and 3c. Mean RTs and MDs are reported in Table 1. RTs were significantly longer in the competitor than distractor condition, *Est*. = −0.05, *SE* = 0.01, *t*(29.16) = −3.39, *p* < .01. MDs were also significantly more attracted to competitors than distractors, *Est*. = −0.11, *SE* = 0.03, *t*(30.41) = −3.49, *p* < .01. Finally, Bayes’ factor analyses provided strong (BF_10_ > 10 for MDs) to decisive (BF_10_ > 100 for RTs) support for these differences (see Table 1). Consistent with Huettig and McQueen (2007) and McQueen and Viebahn (2007), and Experiment 1, these results support the activation of phonological competitors during language processing.

### Experiment 4

Experiment 4 was identical to Experiment 3, but participants heard environmental sounds rather than the spoken words while viewing text arrays. Accuracy was high across competitor (*M* = 99.74%, *SD* = 1.29) and distractor (*M* = 99.74%, *SD* = 1.29) conditions, and 1.54% of trials were above the RT threshold (6.68 seconds). Mean trajectories and deviations are plotted in Figs. 2d and 3d. Mean RTs and MDs are reported in Table 1. Neither RTs, *Est*. = −0.01, *SE* = 0.02, *t*(30.66) = −0.62, *p* = .54, nor MDs, *Est*. = 0.02, *SE* = 0.02, *t*(55.47) = 1.16, *p* = .25, differed significantly between competitor and distractor conditions. A trial-level mixed-effects model assessing the impact of experiment / auditory stimulus type (3 / spoken word = −0.5; 4 / environmental sound = 0.5), visual stimulus type and their interaction on MDs in text arrays also revealed a significant interaction between these fixed effects, *Est*. = 0.13, *SE* = 0.04, *t*(103.62) = 3.59, *p* < .001. Finally, while the Bayes’ factors for MDs provided substantial support (BF_01_ > 3) for the null effect between the competitor and distractor condition, the Bayes’ factors for RTs supported (BF_10_ > 3) a difference (see Table 1). These results hint at a potential weak influence of phonological information on environmental sound processing in the context of text arrays. However, consistent with Experiment 2, this information did not influence participants’ trajectories.

## Discussion

The current experiments revealed a clear divergence between environmental sounds and spoken words. These results provide novel insight into the interaction between nonlinguistic auditory stimuli and linguistic knowledge. Participants’ mouse cursor trajectories were attracted to phonologically related competitors (e.g., belt) during spoken words (e.g., “belt”), conceptually replicating Spivey et al. (2005), but no such phonological competition was observed during environmental sounds (e.g., the ring of a bell). The corresponding Bayes factors for MDs also provided strong to decisive evidence for phonological competition during spoken words, and substantial evidence against such competition during environmental sounds. These results advance understanding of the interaction between nonlinguistic auditory stimuli and linguistic knowledge in two important respects: First, phonological influences on participants’ trajectories were not observed despite the sensitivity of mouse cursor tracking to participants’ continuous behavioural dynamics; and second, no such phonological competition was observed even when participants engaged with visual linguistic contexts (i.e., text arrays). These results support Chen and Spence’s (2011, 2018a) theoretical approach. They hypothesized that environmental sounds are directly mapped onto conceptual knowledge without needing to engage linguistic knowledge, while spoken words are mapped onto phonological representations, which mediate subsequent contact with conceptual knowledge. Correspondingly, participants’ accuracies were at ceiling across both types of auditory stimuli, reflecting clear engagement with conceptual knowledge, but their trajectories only revealed engagement with phonological representations during spoken words and not environmental sounds.

In contrast, these trajectory results are at odds with Bartolotti et al. (2020). They observed fixations to phonologically related competitors (e.g., cloud) during both environmental sounds (e.g., the ticking of a clock) and spoken words (e.g., “clock”). The current experiments were designed to be at least as sensitive; for example, the number of participants (52 vs. 15) and items (30 vs. 15) was greater, and the names of environmental sounds were also normed. Nevertheless, participants’ trajectories diverged markedly between environmental sounds in Experiments 2 and 4 as compared with spoken words in Experiments 1 and 3. These results are compatible with Kukona (2021) and Marian et al. (2021), who likewise observed that environmental sounds did not generate phonological competition. These results also add to their (i.e., eye tracking and recognition) findings by demonstrating related (i.e., null) effects on participants’ continuous behavioural (i.e., mouse cursor) dynamics. Thus, while Bartolotti et al.’s (2020) findings reveal that participants can link nonlinguistic stimuli to phonological representations, we conjecture that the current results, alongside the findings of Kukona (2021) and Marian et al. (2021), also reveal that they can directly activate conceptual knowledge without needing to engage linguistic knowledge at all (the latter may also invite replication of the former).

Consistent with Kukona (2021), we also conjecture that interactions between nonlinguistic stimuli and linguistic knowledge are context dependent. Again, participants in Kukona (2021) fixated phonologically related competitors when participants heard environmental sounds interleaved with spoken words, but not when they only heard environmental sounds. These findings suggest that when participants are engaging with linguistic stimuli, they may also be primed to activate linguistic representations during nonlinguistic stimuli. In contrast, participants only heard environmental sounds in Experiments 2 and 4, and phonological influences were not observed on their trajectories. However, Experiment 4 did require participants to view text rather than picture arrays, and a weak influence was observed on their RTs. Building on Kukona (2021), we conjecture that engaging with text (i.e., reflecting a visual linguistic context) may also (i.e., weakly) prime the activation of phonological relationships during environmental sounds. Relatedly, findings from the visual world paradigm reveal important influences of visual context on the processing of spoken words. For example, Huettig and McQueen (2007) observed differences in the time course of phonological, perceptual and semantic competition that depended on the length of time that participants previewed their visual arrays. While the RT results from Experiment 4 should be interpreted with caution (e.g., they were not significant in the linear mixed effects analysis), they do hint at a potential influence of visual context that invites further study of visual-auditory interactions during the processing of environmental sounds.

A dimension along which the current environmental sounds and spoken words differed, which is typical of the literature, was their durations. Problematically, this difference raises the possibility that participants may have been slower to start their mouse cursor movements in response to environmental sounds because they were significantly longer in duration than spoken words. Moreover, any competition between targets and non-targets may thus have been resolved before doing so, explaining the (i.e., null) trajectory effects in Experiments 2 and 4. To assess this possibility, trial-level log times to initiate mouse cursor movements (i.e., upward movements from participants’ starting coordinates that exceeded 1% of the visual array) were submitted to mixed effects models with fixed effects of experiment / auditory stimulus type, visual stimulus type and their interaction. The analysis of Experiments 1 (competitor *M* = 0.73 seconds, *SD* = 0.43; distractor *M* = 0.75, *SD* = 0.46) and 2 (competitor *M* = 0.58, *SD* = 0.36; distractor *M* = 0.59, *SD* = 0.38) revealed only a marginal effect of experiment / auditory stimulus type, *Est*. = −0.27, *SE* = 0.14, *t*(96.83) = −1.95, *p* = .05, such that initiation times were marginally faster rather than slower for environmental sounds than spoken words (e.g., potentially providing more rather than less opportunity to observe phonological competition during the former), and the analysis of Experiments 3 (competitor *M* = 0.57, *SD* = 0.36; distractor *M* = 0.55, *SD* = 0.35) and 4 (competitor *M* = 0.61, *SD* = 0.42; distractor *M* = 0.57, *SD* = 0.38) did not reveal any significant effects. Rather, as reflected in the increasing deviations from time zero that span Fig. 3a–d, these results suggest that participants’ mouse cursors were in motion during both environmental sounds and spoken works.

Despite many parallels between environmental sounds and spoken words, the literature also reveals important processing differences. For example, Lupyan and Thompson-Schill (2012) found that both environmental sounds and spoken words primed corresponding pictures, but the latter also did so more. They argue that labels are especially effective at activating conceptual representations, consistent with the label feedback hypothesis (Lupyan, 2012). Edmiston and Lupyan (2015) hypothesized that this label advantage emerges because environmental sounds reflect motivated cues, which encode idiosyncrasies about their sources, while labels reflect unmotivated cues, which transcend these idiosyncrasies. For example, the label “bell” can be used to refer to both a bicycle bell and church bell, despite their many differences. Correspondingly, Edmiston and Lupyan (2015) found that participants were slower to verify that pictures (e.g., acoustic guitar) were basic category matches of corresponding environmental sounds (e.g., the strum of an acoustic guitar) as compared with spoken words (e.g., “guitar”), and they were even slower to do so for environmental sounds that were produced by within-category variants (e.g., the strum of an electric guitar). The current results complement this distinction by suggesting that nonlinguistic auditory stimuli do not activate (e.g., unmotivated) labels like spoken words. However, a limitation of the current experiments is that only the semantically related targets (e.g., versus other semantic competitors) were presented alongside the auditory stimuli, and thus the current results do not address the activation of more versus less idiosyncratic semantic representations.

Iordanescu et al. (2011) observed another important processing difference. They found that both environmental sounds and spoken words facilitated visual search for corresponding pictures, but only the latter did so for text. They argue that this difference is grounded in experience, such that both types of auditory stimuli typically co-occur with pictures, but only spoken words typically co-occur with text. Their findings also complement the current results: although (i.e., visual) linguistic and (i.e., auditory) nonlinguistic stimuli were interleaved in Experiment 4, similar to Kukona (2021), this was in the form of text, which may be subject to experiential constraints that yield weaker effects. A related experiential consideration, which builds on classic work by Snodgrass (1984), is that pictures may also activate different aspects of conceptual knowledge than text, potentially contributing to the diverging RT results in Experiments 2 and 4. For example, pictures (e.g., in contrast to text) capture perceptual regularities (e.g., mammals have four legs) that may enable participants to bypass much of conceptual (i.e., not to mention linguistic) knowledge when mapping environmental sounds onto pictures. Again, these RT results should be interpreted with caution, but they do invite further study of these issues.

Finally, the current results also have important methodological implications. The current experiments were “online” in two respects: first, mouse cursor tracking provided a continuous (i.e., online) measure of behaviour throughout processing, contrasting with measures like reaction times and even fixations; and second, data collection was internet mediated (i.e., online), contrasting with lab research. Mouse cursor tracking is growing in prominence (e.g., see Freeman et al., 2011; Schoemann et al., 2021), especially given its sensitivity to continuous behavioural dynamics. However, another advantage is its adaptability to internet-mediated research: participants connecting via computer will typically have a mouse or related device (e.g., trackpad), contrasting with specialized equipment like electroencephalography. On the one hand, internet-mediated research introduces considerable noise. For example, Schoemann et al. (2021) described minimal reporting standards for mouse cursor tracking research that emphasize many design features that simply cannot be controlled and/or known with certainty in internet-mediated research, especially as concern participants’ mouse tracking devices, monitors and software settings (e.g., cursor speed), as well as the physical sizes of the visual stimuli and the distances between them on participants’ monitors. In the current experiments, related information was recorded, including participants’ operating systems and screen resolutions, and these did vary considerably, suggesting that these other features also likely varied. On the other hand, the current results suggest that online mouse cursor tracking is as sensitive to the moment-by-moment dynamics of language processing as lab-based methods (e.g., Allopenna et al., 1998; Huettig & McQueen, 2007; McQueen & Viebahn, 2007; Spivey et al., 2005), transcending this noise. Moreover, beyond mere non-significance or noise, the current Bayes factors also provide evidence against phonological competition during nonlinguistic auditory stimuli, complementing Kukona (2021) and Marian et al. (2021), and providing new insights based on continuous behavioural dynamics.

## Conclusions

The current mouse cursor tracking experiments reveal that phonological representations are not activated by environmental sounds like spoken words. These results suggest that nonlinguistic auditory stimuli directly engage conceptual knowledge without needing to engage linguistic knowledge.

## Data Availability

The data sets generated during and/or analyzed during the current study are available in OSF (10.17605/OSF.IO/ZSJD6). The materials are described in the Appendix.

## Appendix

Targets, competitors and distractors from Experiments 1–4. Pictures in Experiments 1 and 2 were from BOSS (Brodeur et al., 2010; Brodeur et al., 2014); file names are reported. Environmental sounds in Experiments 2 and 4 were from Freesound (https://www.freesound.org); identifiers are reported. Finally, the proportions of participants in the norming whose responses to the environmental sounds agreed with the target names are reported.

**Table 2:** Experimental items

Target	Competitor	Distractor	BOSS T	BOSS C/D	ID	Norm
banjo	bandage	helmet	banjo.jpg	bandage.jpg	533545	0.73
bird	burner	cap	sapsucker.jpg	gasburner.jpg	9328	0.93
camera	camel	pill	camera01a.jpg	dromedary.jpg	51360	0.60
cat	cap	burner	cat.jpg	cap01a.jpg	476485	0.73
chainsaw	chair	windmill	chainsaw.jpg	chair.jpg	413550	0.60
cow	couch	hard hat	cow.jpg	couch02.jpg	58277	1.00
duck	duct tape	chair	duck01.jpg	ducttape.jpg	185134	0.80
hammer	hanger	truck	hammer01.jpg	hanger02a.jpg	406048	0.67
harmonica	hard hat	protractor	harmonica.jpg	toyhardhat.jpg	325381	0.87
helicopter	helmet	bandage	helicopter.jpg	skihelmet01.jpg	322179	0.87
lawn mower	laundry basket	duct tape	lawnmower.jpg	laundrybasket01a.jpg	431478	0.87
pig	pill	couch	pig.jpg	pill.jpg	536746	0.87
printer	protractor	camel	printer02.jpg	protractor.jpg	473830	0.67
trumpet	truck	hanger	trumpet.jpg	boxtruck.jpg	413205	0.60
windshield wiper	windmill	laundry basket	windshieldwiper02.jpg	windmill.jpg	50902	0.80
bell	belt	hose	callbell.jpg	belt02a.jpg	513941	0.73
car	cauliflower	gift	car.jpg	cauliflower01.jpg	186959	0.60
dog	doors	vase	saintbernard.jpg	doubledoors.jpg	24965	0.93
drum set	dresser	visor	drumset.jpg	dresser02.jpg	321435	0.87
elephant	elbow	microscope	africanelephant.jpg	elbow.jpg	139875	0.93
guitar	gift	shovel	acousticguitar02.jpg	gift01.jpg	868	1.00
horse	hose	belt	horse.jpg	hose.jpg	269571	0.87
microwave	microscope	ruler	microwave.jpg	microscope.jpg	508690	0.93
piano	peacock	cauliflower	uprightpiano01.jpg	peacock.jpg	511749	0.93
rooster	ruler	sandal	rooster.jpg	ruler04.jpg	19082	0.93
saxophone	sandal	dresser	saxophone.jpg	sandal.jpg	660	0.73
shower	shovel	peacock	shower.jpg	shovel01.jpg	557831	0.67
vacuum	vase	elbow	vacuumcleaner01.jpg	vase01.jpg	542149	0.73
violin	visor	zebra	violin.jpg	visor.jpg	504678	0.87
zipper	zebra	doors	zipper.jpg	zebra.jpg	6232	0.80

## Code availability

The code used in the analysis of the current study is available in OSF (10.17605/OSF.IO/ZSJD6).
